# Comment on Theeuwes’s Characterization of Visual Selection

**DOI:** 10.5334/joc.29

**Published:** 2018-05-14

**Authors:** Howard Egeth

**Affiliations:** 1 Johns Hopkins University, US

**Keywords:** Attention, Capture, Visual search

## Abstract

Theeuwes (2018, this issue) argues that the classic dichotomy describing the factors that guide attention (bottom-up and top-down) is inadequate and should be replaced by a trichotomy (bottom-up, top-down, and selection history). In contrast, I argue that top-down is a broad category that comfortably includes selection history. While one can certainly choose to subdivide broad categories, there is no obvious stopping point for such an endeavor; how long can it be before this trichotomy turns into a “quadchotomy”?

Theeuwes (2018) presents an updated version of the view of attentional guidance presented by Awh, Belopolsky, and Theeuwes (2012). The main argument of both papers is that the classical analysis of visual selection into top-down and bottom-up components is inadequate; the dichotomy should be replaced by a trichotomy, with the third component being selection history.

Theeuwes (2018) suggests there is fairly broad agreement in the field about the meaning of the phrase bottom-up. Bottom-up attention is driven by the nature of environmental stimuli (e.g., loud sounds, conspicuous feature singletons, and the like). The problem he addresses lies in the meaning of top-down. There is indeed wide agreement here that strategies and plans are top-down. Thus, in Theeuwes’s additional singleton paradigm, if a subject is set to find the one oddball shape in a display of green items, that search is said to be driven by a top-down set for a shape singleton. But, lo and behold, if a single red item is present in such a display there are conditions in which there is a good chance a subject will attend to it first, before moving on to find the distinctive shape. Such a result is often taken to demonstrate the power of the bottom-up component of attention. There are also conditions in which a subject is immune to such capture. That is, “bottom-up” capture may be contingent on some pre-existing mental set (e.g., Bacon & Egeth, 1994; Leber & Egeth, 2006; Folk, Remington, & Johnston, 1992). Thus, Theeuwes may be overly optimistic about just how wide the agreement is about the meaning of “bottom up” (see also Benoni, 2018). We ignore that issue here and will focus on whether the top-down component needs to be subject to further refinement.

Let us imagine now a search for a shape singleton carried out when all of the items in the display are of different colors (so there is no inherent salience advantage for any of the colors), but subjects have been repeatedly rewarded for finding one specific color in a previous phase of the research. It turns out that subjects will tend to attend to the previously rewarded stimulus first, even though now it is not to the subject’s advantage to do so and, in fact responding to the rewarded stimulus is in opposition to the top-down goals of the subject. (e.g., Anderson, Laurent, & Yantis, 2011).

So, how should this interesting reward effect be characterized? I have absolutely no problem calling it a top-down effect. For me, as for many others, top-down is a broad category. Consider, for example the following discussion from a paper on attention in birds. “The selection of the highest priority stimulus for attention is determined by competitive interactions among the neural representations of all stimuli in the environment. Two aspects of each stimulus influence these competitive interactions….: its physical properties, such as its intensity, speed of motion or novelty, and its relevance to the animal’s behavior, such as whether the stimulus predicts reward or whether the animal intends to direct its gaze toward the stimulus (Mysore & Knudsen, 2013, p. 473).” I fail to see anything important being lost in this dichotomous description.

Roughly speaking, I think we have here something analogous to the distinction between perceptual processes (bottom-up) and cognitive processes (top-down). Should things be broken down more finely than perception and cognition? The situation seems comparable to the design of a curriculum for a college or university. Imagine a small school already has in place two courses, one on perception and one on cognition. Let’s imagine that the cognition course includes sections on judgement and decision making, reasoning, creativity, and memory. Depending on the particulars of the school, taking into account both the size of the psychology faculty as well as the size and nature of the student body, that might well be an ideal course organization. However, it’s easy to imagine a new faculty member coming along who feels that it’s not right that the course should include what he sees as the essentially associative faculty of memory being taught along with the parts of the course that are *really* cognitive, such as problem solving. Given the keen interest of this faculty member in memory, maybe it would make sense to offer three courses, perception, cognition, and memory. But as time goes on and still more faculty are added I don’t see any obvious stopping point. The memory course can be further divided in many ways and ever more specific, not to say rarefied, varieties of memory could well become the topics of their own courses, which is, of course, precisely what happens at the graduate level in our largest and most advanced institutions. Importantly, these varieties of memory may well differ from one another in very substantial ways (e.g., they may differ in computational principles or be subserved by different neuroanatomical systems).

The broad point is that there is not necessarily a right or wrong answer to the question of how a field should be divided up. Our personal satisfaction with any particular split may depend as much on our personality, whether we are a “lumper” or a “splitter,” as on the state of nature. In the context of a discussion of a dichotomy prominent in the characterization of alcoholism, Barbor (1994) writes, “What we need is fewer debates between lumpers and splitters, and more attention to better theories and methods.” That comment seems appropriate in the present context as well.

## Data Accessibility Statement

The author has no data accessibility statement to declare.
